# Friction-Induced Mitochondrial Dysregulation Contributes to Joint Deterioration in Prg4 Knockout Mice

**DOI:** 10.3390/ijms18061252

**Published:** 2017-06-11

**Authors:** Kimberly A. Waller, Ling X. Zhang, Gregory D. Jay

**Affiliations:** 1 Department of Orthopedics, Warren Alpert School of Medicine and Rhode Island Hospital, Providence, RI 02903, USA; kimberlyawaller@gmail.com; 2 Department of Emergency Medicine, Warren Alpert School of Medicine and Rhode Island Hospital, Providence, RI 02903, USA; lzhang1@lifespan.org; 3 School of Engineering, Brown University, Providence, RI 02912, USA; 4 Department of Emergency Medicine, Rhode Island Hospital, Coro West, 1 Hoppin Street, Suite 106, Providence, RI 02903, USA

**Keywords:** caspase-3, apoptosis, friction, lubricin, PRG4, chondrocytes

## Abstract

Deficiency of PRG4 (lubricin), the boundary lubricant in mammalian joints, contributes to increased joint friction accompanied by superficial and upper intermediate zone chondrocyte caspase-3 activation, as shown in lubricin*-*null (*Prg*4^−/−^) mice. Caspase-3 activity appears to be reversible upon the restitution of *Prg4* either endogenously in vivo, in a gene trap mouse, or as an applied lubricant in vitro. In this study we show that intra-articular injection of human PRG4 in vivo in *Prg*4^−/−^ mice prevented caspase-3 activation in superficial zone chondrocytes and was associated with a modest decrease in whole joint friction measured ex vivo using a joint pendulum method. Non-lubricated *Prg*4^−/−^ mouse cartilage shows caspase cascade activation caused by mitochondrial dysregulation, and significantly higher levels of peroxynitrite (ONOO^−^ and ^−^OH) and superoxide (O^−^_2_) compared to *Prg*4^+/+^ and *Prg*4^+/−^ cartilage. Enzymatic activity levels of caspase 8 across *Prg*4 mutant mice were not significantly different, indicating no extrinsic apoptosis pathway activation. Western blots showed caspase-3 and 9 activation in *Prg*4^−/−^ tissue extracts, and the appearance of nitrosylated Cys163 in the active cleft of caspase-3 which inhibits its enzymatic activity. These findings are relevant to patients at risk for arthrosis, from camptodactyl-arthropathy-coxa vara-pericarditis (CACP) syndrome and transient lubricin insufficiency due to trauma and inflammation.

## 1. Introduction

Deficiency of lubricin (PRG4), the principal boundary lubricant in cartilage, contributes to progressive joint failure that is hallmarked by increased joint friction, superficial and upper intermediate zone apoptosis, and synovial hyperplasia, as shown in *Prg*4-null mice [1] and patients with camptodactyl-arthropathy-coxa vara-pericarditis (CACP) syndrome [2,3]. Lubricin-null (*Prg*4^−/−^) mice joints appear normal at birth but soon exhibit significant joint degradation, compared to heterozygous (*Prg*4^+/−^) and wild-type (*Prg*4^+/+^) littermates, which express lubricin [4,5]. Patients with CACP have used non-steroidal anti-inflammatory medications to seek symptom relief, which suggests that inflammation may play a role in the lubricin-null joint pathology that hitherto was thought to be a non-inflammatory arthropathy. In the absence of disease-modifying treatments, besides total joint replacement, lubricin delivered by intra-articular injection [6,7] may be a viable episodic treatment for the joints of lubricin-null individuals. Cartilage surfaces of *Prg*4^−/−^ mice are biofouled with albumin and other synovial fluid non-lubricating constituents [4]. In the absence of lubricin, *Prg*4*^−/−^* joints may experience inflammation due to elevated friction. We theorized that an elevation in reactive oxygen species (ROS) originating from the chondrocyte mitochondria, contribute to cellular dysfunction and is caused by elevated articular cartilage friction which promotes exaggerated shear deformation upon ambulation. Thus, intra-articular injection of lubricin would reduce cellular dysfunction and apoptosis as reflected by lower caspase-3 activation in lubricin-null mice treated with purified lubricin.

Loss of superficial zone cellularity is well documented in *Prg*4^−/−^ mice [8] in which the chondrocytes display caspase-3 activation. In *Prg*4^GT^ mice, which are born lubricin-null, the early restitution of *Prg4* expression at 3 weeks appears to suppress caspase-3 activation and partially restore low friction [1]. However, the joints of both younger and older lubricin-null mice do not show full-thickness degeneration despite the large numbers of chondrocytes displaying caspase-3 activation. These observations suggest that a process is in play that could inhibit intrinsic apoptosis that is also tribologically dependent. As a result, we posited that the intra-articular administration of lubricin, which is effective in preventing degeneration in trauma-induced preclinical rodent models [6,9,10,11,12], may also be effective in lubricin-null mice; the orthologous genetic model of CACP syndrome in humans.

We hypothesized that the supplementation of purified human synoviocyte lubricin (HSL) into the joint cavity of *Prg*4^−/−^ mice at 6-weeks of age would lower whole joint coefficient of friction (COF) in vivo and prevent chondrocyte apoptosis, compared to the contralateral joint, measured at 2 weeks following injection and compared to age-matched littermates that did not receive treatment. To this end, we measured inflammatory cytokines, ROS production and caspase-3 expression on the mRNA and protein levels in the cartilage and synovium of *Prg*4^−/−^ and *Prg*4^+/+^ mice. We also measured the presence of the neoepitope caused by the nitrosylation of Cys163 in caspase-3, which is inhibitory. These studies are relevant to patients with CACP syndrome and others with transient loss of PRG4 in inflammatory joint diseases such as traumatic injuries which are known to place a patient at risk for osteoarthritis (OA).

## 2. Results

### 2.1. Whole Joint COF of Prg4^−/−^ Mice Following Intra-Articular PRG4 Compared to Sham Injected and Control Mice

**Figure 1:**
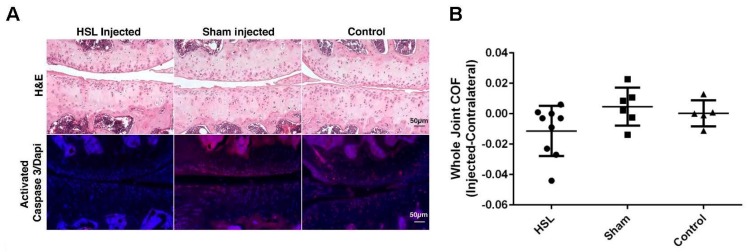

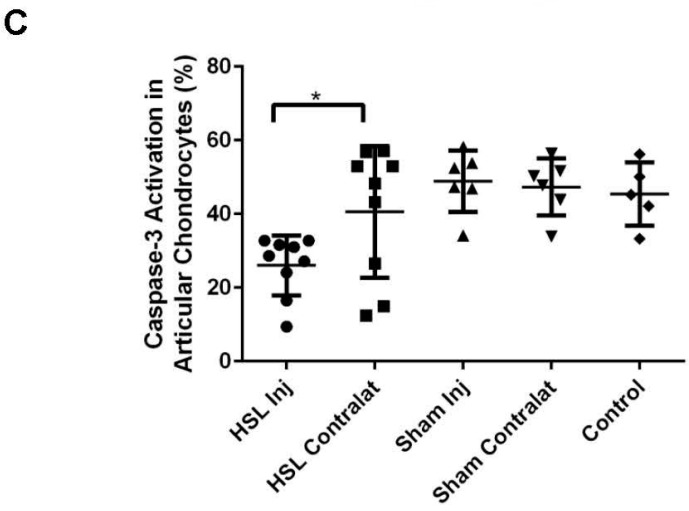
Lubricin-null *(Prg*4^−/−^) mouse tibio-femoral joints following intra-articular (IA) injection in vivo with purified human synoviocyte lubricin (HSL; ●): (**A**) Hematoxylin and eosin stained cartilage shows damage in joints treated and untreated with intra-articular HSL (PRG4). Sub-surface chondrocytes displaying caspase-3 activation are noted in *Prg*4^−/−^ mice, which appear decreased 14 days following intra-articular PRG4. In another group of age matched sham injected *Prg*4^−/−^ mice the qualitative number of chondrocytes displaying caspase-3 activation counterstained with 4,6-diamidino-2-phenylindole, dihydrochloride (DAPI) is not different from control *Prg*4^−/−^ tibio-femoral joints. The untreated contralateral joints (*n* = 9) in PRG4-treated mice qualitatively show more caspase-3 activation in sub-surface chondrocytes (scale bar is 50 μm); (**B**) Box plots showing partial restoration of whole joint coefficient of friction (COF) in *Prg*4^−/−^ mice treated with IA HSL (●), phosphate buffered saline (PBS; ■) or no injection (control; ▲) ; (**C**) Box plots of the same joints show a significant decrease in the percentage of chondrocytes with detectable caspase-3 activation following IA HSL (●) compared to the contralateral joints (■), * *p* = 0.024, IA PBS (▲), IA PBS contralateral joints (▼) or no injection (control; ◆).

Based on analysis of variance, differences in COF between injected and contralateral knee joints were not significantly different across the three experimental conditions (*p* = 0.096) and all joints showed damage to menisci and articular cartilage (Figure 1A). However, within-group analyses indicated a significantly lower mean COF in HSL-treated *Prg*4^−/−^ knee joints compared to their contralateral joints (*p* = 0.024) while sham injected and control mice did not show differences in COF on comparison with their contralateral joints (*p* = 0.42 and 0.96 respectively) (Figure 1B).

### 2.2. Caspase-3 Activation of Prg4^−/−^ Mice Following Intra-Articular PRG4

Based on analysis of variance, differences in the percentage of chondrocytes displaying caspase-3 activation between injected and contralateral knee joints were not significantly different across HSL-treated and sham-treated conditions (*p* = 0.06) (Figure 1C). However, within-group analyses indicated a significantly lower percentage of chondrocytes displaying caspase-3 activation in HSL-treated *Prg*4*^−/−^* knee joints compared to their contralateral joints (*p* = 0.024) while sham injected *Prg*4^−/−^ mice did not show a difference in caspase-3 activation when compared to their contralateral joints (*p* = 0.80). The background level of caspase-3 activation in non-injected control animals was similar to sham injected mice (*p* = 0.51).

### 2.3. Peroxynitrite (PN) Assay in Prg4 Mutant Mice Femoral Head Cartilage

**Figure 2:**
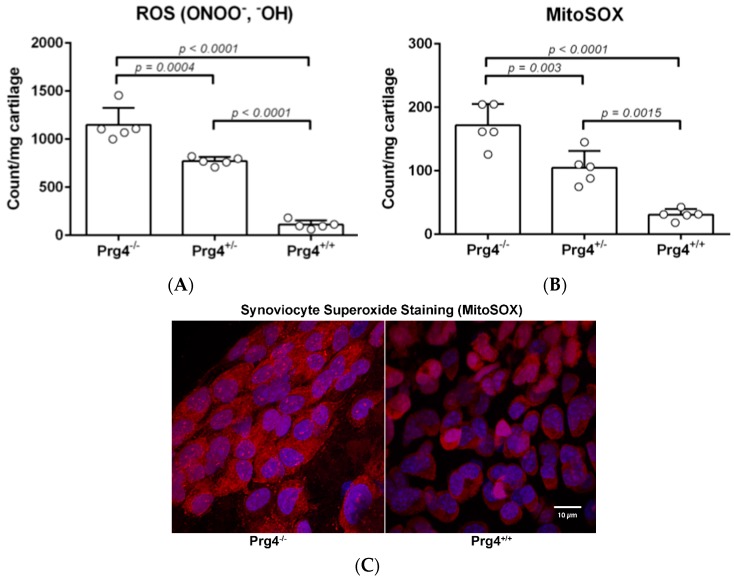
Inflammation in *Prg4* mutant mice: (**A**) Peroxynitrite (ONOO^−^ and ^−^OH) and (**B**) mitochondrial superoxide (MitoSOX production in resected femoral heads from 10-wk-old wild-type (*Prg*4^+/+^), heterozygous lubricin knockout (*Prg*4^+/−^), and homozygous lubricin knockout (*Prg*4^−/−^) mice. Prg4-insufficient cartilage showed a significantly higher level of both the reactive oxygen species (ROS) ONOO^−^ and OH·, as well as MitoSOX, as compared to *Prg*4^+/−^ and *Prg*4^+/+^ cartilage. ROS were significantly lower in *Prg*4^+/−^ cartilage compared to *Prg*4^−/−^ cartilage, but were significantly higher than in wild-type *Prg*4^+/+^ cartilage; (**C**) Levels of superoxide, detected by MitoSOX (red) and counterstained with DAPI, are greater in *Prg*4^−/−^ synoviocytes as compared to *Prg*4^+/+^ counterparts. *Prg*4^−/−^ synoviocytes also show presence of superoxide in the cytoplasmic and extracellular matrix space (scale bar is 10 μm).

Tissue weight normalized fluorescence values for PN (ONOO^−^ and OH·) generated by resected femoral head cartilage from *Prg*4 mutant mice showed that *Prg*4^−/−^ cartilage generated significantly more PN as compared to *Prg*4^+/−^ (*p* < 0.001) and *Prg*4^+/+^ (*p* < 0.0001) cartilage (Figure 2A). A comparison of the PN levels between femoral head cartilage from *Prg*4^+/−^ and *Prg*4^+/+^ littermates also showed a significant difference (*p* < 0.0001). Peroxynitrite is a mitochondrial metabolite formed from superoxide ($O_{2}^{-}$) and nitric oxide (NO).

### 2.4. Superoxide (MitoSOX) Assay in Prg4 Mutant Mice Synoviocytes

We observed significantly higher levels of mitochondrial superoxide produced by *Prg*4*^−/−^* synoviocytes compared to synoviocytes from *Prg*4*^+/+^* (*p* < 0.0001) and *Prg*4*^+/−^* (*p* = 0.0004) littermates (Figure 2B). *Prg*4*^+/−^* mice had significantly higher levels of superoxide compared to wild-type mice (*p* < 0.0001). *Prg*4*^−/−^* mice had significantly higher levels of superoxide compared to *Prg*4*^+/+^* (*p* < 0.00001) and *Prg*4*^+/−^* mice (*p* = 0.003) which was also visualized by fluorescent microscopy where the cytoplasm of *Prg*4*^−^*^/−^ synoviocytes qualitatively contained much more superoxide (Figure 2C). *Prg*4*^+/−^* mice had significantly higher levels of superoxide compared to wild-type mice (*p* = 0.0015).

### 2.5. Caspase-8 Activity in Chondrocytes and Synoviocytes from Prg4 Mutant Mice

Isolated chondrocytes and synoviocytes from *Prg*4^−/−^ mice did not show a statistically greater amount of caspase-8 activity units compared to *Prg*4^+/−^ and *Prg*4^+/+^ littermates (*p* > 0.05). Background activity was 39.30 ± 1.53 activity units. Activity for *Prg*4^−/−^ chondrocytes was 120.67 ± 1.17 compared to 113.71 ± 1.03 for *Prg*4^+/−^ and 113.91 ± 2.99 for *Prg*4^+/+^ chondrocytes. Activity for *Prg*4^−/−^ synoviocytes was 130.09 ± 1.85 compared to 129.48 ± 1.24 for *Prg*4^+/−^ and 124.58 ± 3.16 for *Prg*4^+/+^ synoviocytes. This indicates that extrinsic apoptosis pathways are not active in *Prg*4 mutant chondrocytes and synoviocytes.

### 2.6. Real Time PCR of mRNA Recovered from Cartilage of Prg4 Mutant Mouse Joints

**Figure 3:**
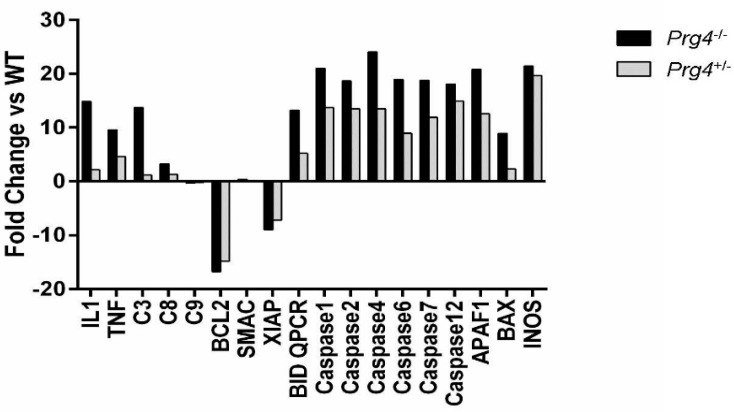
Real-time PCR of intrinsic and extrinsic apoptosis mediators from chondrocytes of knee articular cartilage from three *Prg*4^−/−^ and *Prg*4^+/−^ mice. The ∆∆*C*_t_ calculation represented as a log2 fold change relative to the *Prg*4^+/+^ genotype is shown. Levels of caspase-3 and IL-1β expression are greater in chondrocytes from *Prg*4^−/−^ than *Prg*4^+/−^ littermates, however expression levels of caspase-8 and 9 are unchanged. Levels of factors involved in intrinsic apoptosis, including *Bax*, BH3 interacting-domain death agonist (*Bid*) and Caspase-2 all show greater expression in *Prg*4^−/−^ as compared to *Prg*4^+/−^ chondrocytes. Both of these genotypes show significant downregulation of B-cell lymphoma 2 (*Bcl-2*) which stabilizes the mitochondrial membrane, preventing the release of cytochrome C, and X-linked inhibitor of apoptosis protein (*XIAP*) which inhibits the activated executioner caspase-3 and caspase-9. This suggests that friction caused by lubricin insufficiency through the *Prg*4^−/−^ null state or mild insufficiency in the *Prg*4^+/−^ heterozygous state induces mitochondrial stress.

We observed significantly higher expression of *IL-1β* and *TNF-α* in the cartilage of *Prg*4*^−/−^* mice compared to *Prg*4*^+/+^* (*p* < 0.0001) and *Prg*4*^+/−^* (*p* < 0.0001) mice, and in *Prg*4*^+/−^* mice compared to *Prg*4*^+/+^* mice (*p* < 0.0001) (Figure 3). Elevated expression of caspase 3, 6 and 7 occurred in *Prg*4*^−/−^* mouse cartilage, indicating intrinsic apoptotic pathway activity relating to mitochondrial involvement. Expression of caspase 9 was unchanged, while expression of caspase 8 was only slightly increased in the cartilage of *Prg*4^−/−^ mice relative to *Prg*4^+/+^ mice. Most importantly, the expression of B-cell lymphoma 2 (*Bcl-2*) and X-linked inhibitor of apoptosis protein (*XIAP*) was significantly downregulated in cartilage from *Prg*4^−/−^ and *Prg*4^+/−^ relative to *Prg*4^+/+^ mice.

### 2.7. Western Blots and Densitometry

**Figure 4:**
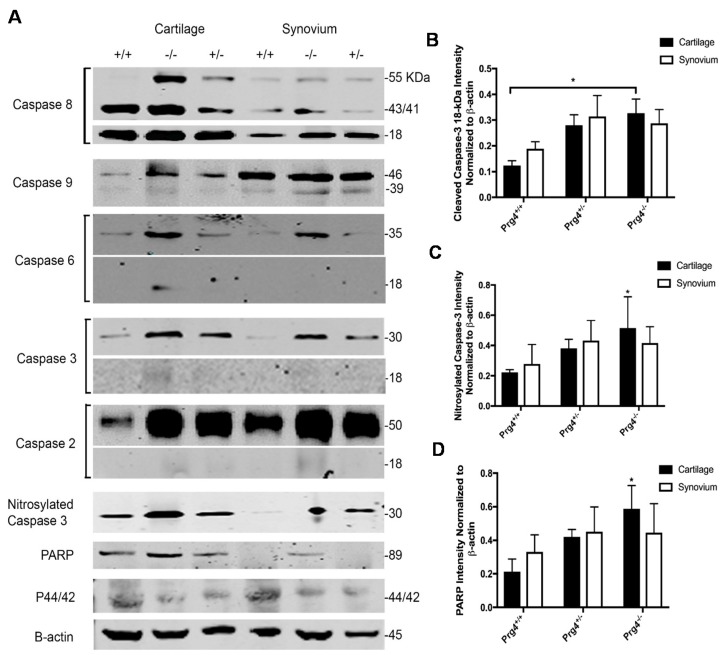
Western blots and densitometry of apoptosis mediators from *Prg*4 mutant mice: (**A**) Montage of western blots of extrinsic apoptosis pathway factor (caspase 8), intrinsic pathway factors (caspase 2, 6 and 9), caspase 3, nitrosylated Cys163 neo-epitope of caspase 3, cleaved poly (ADP-ribose) polymerase (PARP) and p44/42 across cartilage and synovium of five *Prg*4*^−/−^*, *Prg*4*^+/−^* and *Prg*4*^+/+^* mice. Bands were scanned and normalized to β-actin in the same lanes; (**B**) Densitometry of the cleaved caspase-3 18-kDa band; (**C**) Nitrosylated Cys163 neo-epitope of caspase 3; (**D**) Cleaved PARP across all three *Prg*4 genotypes. More apoptosis mediators illustrated in (**B**–**D**) were detected in cartilage from *Prg*4^−/−^ mice than in *Prg*4^+/+^ littermates, * *p* < 0.05.

Qualitatively, there was a greater amount of phosphorylated MAP kinase (p44/42) in both *Prg*4^+/+^ chondrocytes and synoviocytes compared to *Prg*4^+/−^ and *Prg*4^−/−^ littermates (Figure 4A). There was no difference in the 18-kDa pro-apoptotic fragment from caspase 8 cleavage within chondrocytes across the three genotypes, and no apparent difference for the 43-kDa caspase 8 intermediate fragment between the *Prg*4^+/+^ and *Prg*4^−/−^ genotypes (Figure 4A). There were more cleaved caspase 3 18-kDa fragments in *Prg*4^−/−^ chondrocytes and no differences for synoviocytes across the three genotypes (Figure 4B). The neoepitope formed by the inhibitory nitrosylation of residue Cys163 in the active cleft of caspase-3 was in greater abundance in *Prg*4^−/−^ mice as compared to *Prg*4^+/+^ littermates (*p* < 0.05) and as opposed to *Prg*4^+/−^ mice (*p* = 0.98) (Figure 4C). Band densities for the nitrosylated-Cys163 neoepitope for *Prg*4^+/−^ and *Prg*4^+/+^ were not significantly different. More cleaved poly (ADP-ribose) polymerase (PARP) was present in chondrocytes from *Prg*4^−/−^ mice as compared to *Prg*4^+/+^ littermates (*p* = 0.03) and as opposed to *Prg*4^+/−^ (*p* = 0.55) mice. However, band densities for *Prg*4^+/−^ and *Prg*4^+/+^ were not significantly different from each other (Figure 4D).

### 2.8. Immunohistochemistry of Nitrosyl-Cys163 Caspase-3 Neoepitope in Cartilage from Prg4^−/−^ Mouse Joints

**Figure 5:**
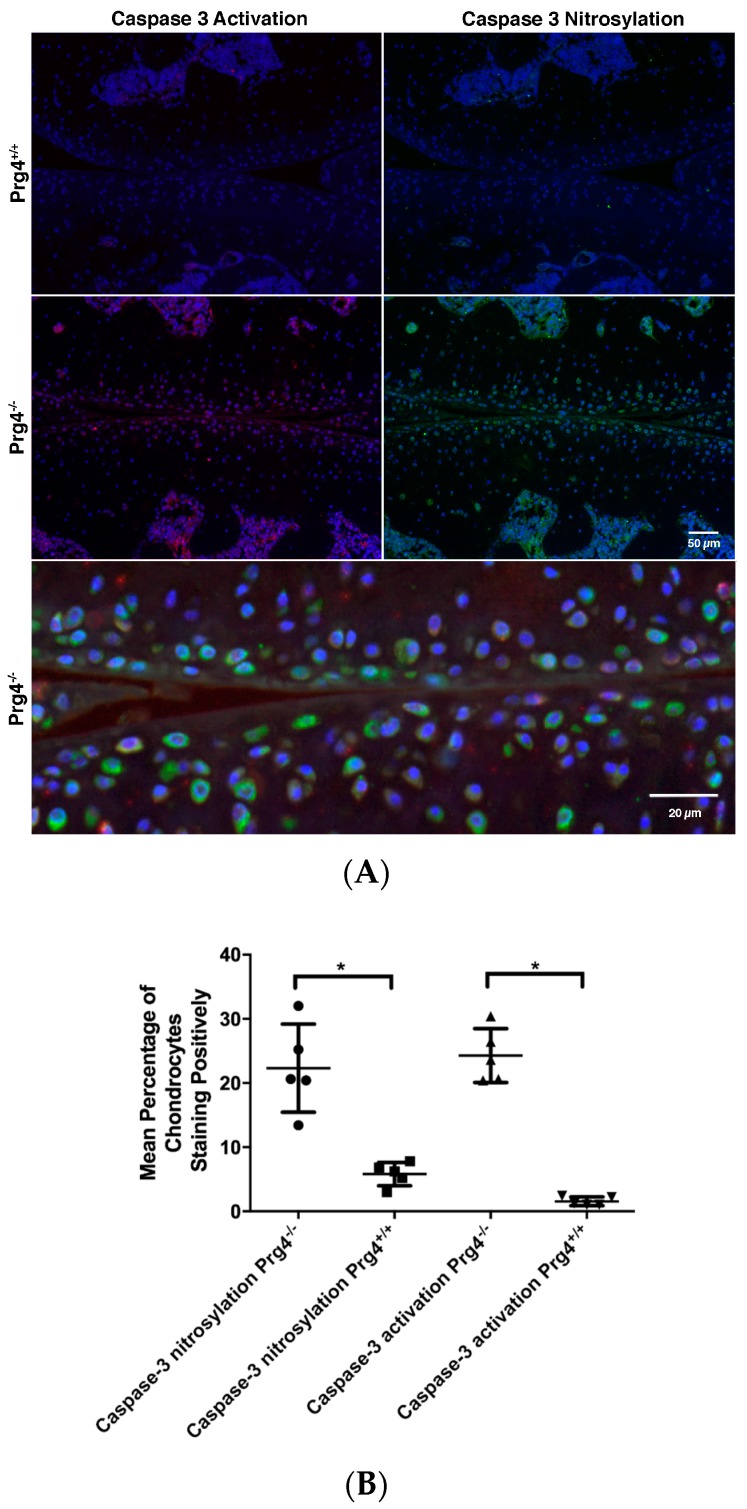
Immunohistochemistry of tibio-femoral joints from *Prg*4^−/−^ and *Prg*4^+/+^ mice for caspase-3 activation and nitrosylated Cys163 of caspase-3: (**A**) Most immunopositivity of chondrocytes is located just beneath the articular surface. Both immunoprobes competed for adjacent epitopes within the active cleft of caspase-3 which upon dual probing merged both immunofluorescent stains within chondrocytes (scale bar is 20 μm); (**B**) Semi-automated counting of DAPI counterstained chondrocytes from the cartilage surface to the tide mark revealed significantly higher numbers of immunopositive cells for activated caspase-3 (▲) and caspase-3 nitrosylation (●) in *Prg*4^−/−^ as compared to *Prg*4^+/+^ cartilage respectively (▼, ■ ), * *p* < 0.05 (scale bar is 50 μm).

Most chondrocytes that were immuno-positive were located just beneath the articular surface (Figure 5A). Counting and analysis spanned from the articular surface to the tide mark. The percentage of chondrocytes displaying caspase-3 activation was 24.3 ± 4.2 in *Prg*4^−/−^ knee joints, which exceeded 1.6 ± 0.7 (*p* < 0.05) in *Prg*4^+/+^ knee joints (Figure 5B). The percentage of chondrocytes displaying the nitrosyl-Cys163 caspase-3 neoepitope in chondrocytes was 22.3 ± 6.8 in *Prg*4^−/−^ knee joints, which exceeded 5.8 ± 1.8 (*p* < 0.05) in *Prg*4^+/+^ knee joints (Figure 5B). There was no statistical difference in the percentage of chondrocytes that were positive for caspase 3 activation and in the nitrosyl-Cys163 caspase-3 neoepitopes within *Prg*4^−/−^ cartilage (*p* = 0.41). Dual staining with both probes was not possible since one probe was exclusive of the other regardless of incubation order, suggesting that both probes competed with one another. However, in some instances dual staining was achieved, which revealed co-localization of both the anti-caspase 3 and anti-*S*-nitrosocysteine probes, and 4,6-diamidino-2-phenylindole, dihydrochloride (DAPI) staining within chondrocytes (Figure 5A).

## 3. Discussion

Articular cartilage is highly organized, and built to withstand high shear stress along the superficial zone, and compressive stress through the deeper regions. Chondrocyte phenotype is location-dependent and reflects this organization as flattened chondrocytes in the superficial zone tangentially aligned with collagen type II fibrils [5,13]. Previously, we have shown that rounded chondrocytes in the upper middle zone are at the highest risk for apoptosis in the presence of elevated friction [14]. As the cartilage is compressed, interstitial fluid supports load [15] and provides lubrication in concert with synovial fluid [16] which contains lubricin. The extracellular matrix deforms cells and intracellular mechano-signal transduction relies on the MAPK pathway to transduce mechanical forces into a biological response [17]. Cartilage compression also alerts mitochondria within chondrocytes via the cytoskeleton [18] and mitochondrial dysfunction has been linked to osteoarthritis [19].

In normal joints, lubricin acts as a boundary lubricant and an anti-inflammatory factor, reducing cartilage deformation via lubrication [20], and binds to CD44 to prevent the expression of IL-1β and TNF-α [21], which contribute to synovial overgrowth. Physiological loading and lubricated movement of apposed cartilage surfaces promotes lubricin expression both in vitro [22,23,24] and in vivo [25]. While normal joint loading is required for maintaining cartilage health [26,27], abnormal loading, either due to blunt trauma [28,29], strenuous exercise or friction vis-a-vis the absence of lubricin, leads to chondrocyte apoptosis and osteoarthrosis [14,30]. However, the reconstitution of lubrication in vitro [14] and endogenously [1] leads to a decrease in caspase-3 activation, indicating that the cascade of caspase activation in chondrocytes is either reversible or can be inhibited.

**Figure 6:**
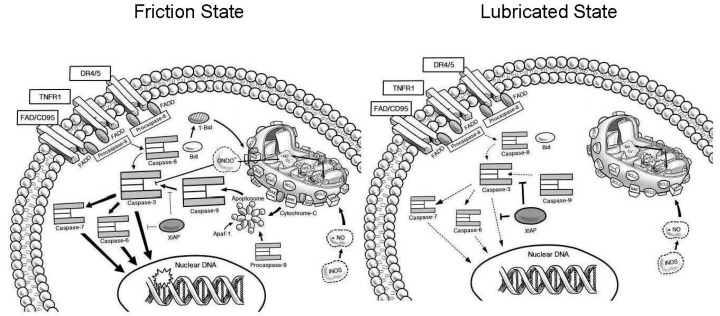
Proposed model of friction-induced mitochondrial dysregulation. A significant increase in the number of chondrocytes that display activation of caspase-3 is caused by friction at the articular cartilage surface from the absence of PRG4. Mitochondrial dysfunction is caused by excessive mechanical strain applied to cartilage tissue and by extension, its embedded chondrocytes. The increase in mitochondrial permeability causes the release of cytochrome C which binds to apoptotic protease activating factor 1 (Apaf-1), resulting in the activation of caspase-9, denoted by bold curved solid arrow, and the caspase-3, 6 and 7 cascade, denoted by bold solid straight arrows, which culminates in intrinsic apoptosis. Levels of Bcl2 are depressed in the high friction state, and unable to counteract Bax and t-Bid, denoted by a bold curved arrow, in promoting mitochondrial membrane permeability. Caspase-3 activation by activated capase-9 is normally inhibited by XIAP in the lubricated state, denoted by the bold blunted solid line, which has lower expression in the friction state resulting in unopposed activation of caspase-3. Although many chondrocytes will show immunohistochemical evidence of caspase-3 activation, these cells do not die and the activation of the intrinsic apoptosis pathway is reversible upon the application of lubricin to the articular surface. Extrinsic apoptosis is not active as friction-induced changes in caspase-8 activity do not occur, and do not initiate the caspase-3, 6 and 7 cascade. Caspase-3 is directly inhibited in the high friction state by peroxynitrite (ONOO^−^), a product of mitochondrial stress, which causes the nitrosylation of Cys163 in the active cleft of caspase-3, denoted by the blunted line. This inhibitory protein modification serves to arrest the progression of intrinsic apoptosis that would otherwise result in caspase-3, 6 and 7 mediated cleavage of PARP and unapposed DNA-fragmentation caused by endonucleases.

Chondrocyte apoptosis can be initiated via several pathways, both intrinsic and extrinsic [31]. Based on our results, we conclude that the primary mechanism in lubricin-null mice is friction-related, and, therefore, intrinsic (Figure 6). In cartilage from *Prg*4*^−/−^* mice, we observed elevated expression of executioner caspases 3 and 6, coupled with cleavage of PARP, as well as cleavage of caspases 3, 6 and 9. Caspase 9 is activated via the mitochondria, when cytochrome-c diffuses across the mitochondrial membrane, following an unbalanced Bcl-2/Bax ratio [32,33]. *Prg*4^−/−^ cartilage exhibited a pronounced decrease in expression of protective Bcl-2, compared to *Prg*4^+/−^ cartilage. Bcl-2 acts as an anti-apoptotic factor in the mitochondrial by binding with pro-apoptotic factor Bax, to reduce permeability of the mitochondria.

The uncleaved form of caspase 8 was elevated in chondrocytes and synoviocytes as detected by Western blot, but the cleaved intermediate 43-kDa fragment and pro-apoptotic 18-kDa fragment were equally prominent for *Prg*4^−/−^ and *Prg*4^+/+^ chondrocytes. Direct assay for caspase 8 activity in these tissues failed to show a significant increase in *Prg*4^−/−^ cartilage compared to *Prg*4^+/−^ and *Prg*4^+/+^ littermates. Autocrine and paracrine effects from the elevated level of TNFα in *Prg*4^−/−^ tissue upon the TNFR1 receptor would normally stimulate caspase 8 cleavage, which was not observed in the recovered chondrocytes. Furthermore, *Prg*4*^+/+^* mice showed elevated p-Erk1/2 in both chondrocytes and synoviocytes, which indicates the presence of survival factors [34] and maintenance of a differentiated chondrocyte phenotype [35] that appears to be lacking in the *Prg*4^−/−^ cartilage.

Mitochondria regulate cell function and survival, and their dysfunction affects several pathways involved in chondrocyte death, which results in the production of NO, reduction in matrix synthesis and apoptosis [36]. The presence of ROS and inflammatory factors in *Prg*4^−/−^ mice would support the catabolic role ROS play in inducing inflammatory cytokines, such as IL-1β and TNF-α, and in compromising the viscoelasticity of cartilage by affecting the proteoglycan–collagen network surrounding chondrocytes [37]. Others have identified excessive mechanical loading as a cause of ROS release [38] and oxidative stress in cartilage senescence [39] in the development of osteoarthritis. Oxidant release is strain-dependent [18] which supports our observations of friction-induced ROS production. However prior reports also indicate that NO is also involved in the nitrosylation of Cys163 in the active cleft of caspase-3 [40]. This neoepitope antagonizes caspase-3 activity and thus NO can play an anabolic role and potentially limit damage to DNA. We detected a significant elevation of this neoepitope in *Prg*4^−/−^ mice by Western blot, however PARP activity was also higher. Thus, the inhibition of caspase-3 may provide an explanation for the continued presence of chondrocytes displaying immunodetectable caspase-3 activation in cartilage from both young and older *Prg*4^−/−^ mice. This is despite high levels of apoptotic factors and PN, which is a derivative of NO, and itself involved in the single-strand breakage of DNA [41] and induction of COX-2 and PGE2 [42].

Elevated levels of PN may provide a protective role in low concentrations, as seen in *Prg*4*^+/−^* cartilage where PN levels are higher than in wild-type littermates, in the absence of either significant elevation in friction or caspase-3 activation [14]. Heterozygous mice appear phenotypically and histologically normal [5,14], but following 18 h of exercise ex vivo, the whole joint COF is elevated to the level of *Prg*4*^−/−^* mice [43] and may be indicative of incomplete chondroprotection. Peroxynitrite component ONOO^−^ plays a role in inflammatory hyperalgesia [44] by sensitizing afferent nociceptive neurons [42] in the joint capsule and synovium [45]. Thus, the presence of PN may serve to lower applied mechanical loads due to locomotion through mechanical allodynia as a direct result of inadequate joint lubrication. Furthermore, active caspase-6 appeared lower in Prg4-expressing mice and caspase-2 expression appeared qualitatively higher in *Prg*4^−/−^ chondrocytes and synoviocytes. Caspase-2 is a known cellular sensor for ROS and is involved in limiting oxidative damage [46].

These findings should be interpreted in the context of recent important studies showing how primary chondrocyte death does not cause an OA phenotype. Killing of 50% of chondrocytes endogenously by diphtheria toxin expression under control of Cre did not cause cartilage degeneration as late as 8 months later [47]. In contrast, chondrocyte deficient littermates that underwent destabilization of the medial meniscus showed ample evidence of cartilage degeneration. Inflammation-dependent downregulation of PRG4 due to IL-1β [24] also occurs in traumatic injuries [7,48] and places the cartilage at risk for chondrocyte demise via friction-induced exaggerated mechanical shear of cartilage that may also deform chondrocyte mitochondria. The resulting cellular stress mechanistically causes intrinsic apoptotic pathway activation and cell death if not corrected by reducing surface friction [14].

Limitations to this study include the need to pool tissues from multiple mice, due the small volume of tissue available via harvest from each animal. Likewise, cartilage may not have been fully isolated from subchondral bone in the PN assays to ensure evaluation of the maximum amount of cartilage. Caspase 3 was not purified from cellular extracts to show that it was chemically inhibited by nitrosylation from ONOO^−^. This study relies on the comparison of mutant *Prg4* mice with wild type littermates that show metabolic differences due to elevated joint friction. The possible arrest of chondrocyte demise by lubricating articular surfaces may provide an early treatment option for major joints in patients with CACP, who show osteoarthritis-like symptoms at an early age. Our group continues to explore if lubricin injection therapy can supplement lubricin insufficiency and forestall or prevent arthrosis. 

## 4. Materials and Methods

### 4.1. Prg4 Mutant Mice

Knee and hip joints from *Prg*4*^+/+^, Prg*4*^+/−^,* and *Prg*4*^−/−^* mice were harvested following euthanasia using asphyxiation with CO_2_ and cervical dislocation. Cartilage was obtained from the tibial plateau and femoral condyle of knee joints. Femoral head cartilage was also collected from hip joints in order to maximize collected cartilage. Synovium was isolated from the knee joints. All animal experiments were approved by the Rhode Island Hospital Animal Welfare Committee under #0199-12 on 25 September 2015. Animals were housed in an AAALAC-accredited facility in accordance with the National Research Council’s Guide for the Care and Use of Laboratory Animals and the Public Health Service Policy on Humane Care and Use of Laboratory Animals.

### 4.2. Tribosupplementation In Vivo of Prg4^−/−^ Mouse Knees

*Prg*4^−/−^ (*n* = 15) mice 6 weeks of age were anesthetized under isoflurane (3–5%). The right hind limb was clipped of hair and the site of injection was prepared with a three-stage preparation involving a povidone iodine scrub, a 70% alcohol wash, and povidone iodine solution. Mice were randomized to either intra-articular (IA) human synoviocyte lubricin (HSL) (*n* = 9) or IA phosphate buffered saline (PBS) (*n* = 6). The knee was held in flexion and 20 µL of sterile HSL (1.6 mg/kg) or PBS was injected via a 31G needle through the patellar tendon, which was confirmed by bulging of the joint space. The needle was removed, and the knee flexed and extended 10 times to ensure even distribution throughout the joint cavity. The contralateral knee was not treated. Injection technique was tested on cadaver mice using 10% India ink dye in water. Two weeks following initial lubricin injection, at 8 weeks of age, injected mice and untreated littermates were euthanized. Hind limbs of the mice were removed for measurement of coefficient of friction.

### 4.3. Whole Joint Coefficient of Friction (COF) Measurement

The hind limbs of the mice were isolated, and tissue surrounding the joint capsule was removed, leaving the joint capsule intact. The distal tibia and proximal femur were rigidly mounted in square brass tubing using urethane glue. Whole joint COF was measured using a modified Stanton pendulum system, as previously described [14,43,49]. Friction measurements were performed with a pendulum load of 50 g, which corresponds to 2 times body weight. Limbs were loaded 8 min prior to each set of measurements to allow cartilage surface apposition and depressurization [50]. Four measurements were taken at 15-min intervals per limb. The equilibrium whole joint COF value at 60 min was used in analyses.

### 4.4. Tissue Harvest for Cells, Protein and mRNA

Messenger RNA (mRNA) and protein was isolated from mouse knee cartilage (*n* = 5 for each genotype) or synovium (*n* = 5 per genotype) and pooled by genotype prior to qRT-PCR and Western blot analysis, respectively. Recovered tissues were also cultured in 96-well cell-culture plates (8 × 10^4^ per well) with Dulbecco’s modified eagle’s medium (DMEM) control for later ROS and caspase-8 analyses.

### 4.5. Quantitative Real-Time Polymerase Chain Reaction

Mouse *IL-1β* and *TNF-α* expression were measured by QPCR. *GAPDH* was used as a housekeeping gene for cartilage, and β-actin for synovium. Additional genes of interest included *caspases 1–4, 6–9* and *12*, *Bcl-2*, *SMAC*, *XIAP*, *Bid*, *APAF1*, *Bax* and *iNOS*. Data is reported using the ∆∆*C*_T_ method. Suitability of *GAPDH* as an internal control gene was tested by calculating $2^{-C_{T}}$ for each of the three mice from each genotype. The fold changes in expression of GAPDH in *Prg*4^−/−^, *Prg*4^+/−^ and *Prg*4^+/+^ relative to each other were calculated separately as $2^{-C_{T}}$(*Prg*4^−/−^)/$2^{-C_{T}}$(*Prg*4^+/+^), $2^{-C_{T}}$(*Prg*4^+/−^)/$2^{-C_{T}}$ (*Prg*4^+/+^) and $2^{-C_{T}}$(*Prg*4^−/−^)/$2^{-C_{T}}$(*Prg*4^+/−^). An a priori fold change near 1 and significantly less than 2 was necessary for internal control gene acceptability. The *C*_T_ from the PCR’s for the target gene and *GAPDH* as the internal control gene for the three animals of each genotype were subtracted and used in the following ∆*C*_T_ equation $2^{-(C_{T\;\mathrm{gene}\;\mathrm{of}\;\mathrm{interest}\;}-C_{T\;\mathrm{internal}\;\mathrm{reference}})}$. Mean ∆*C*_T_ was calculated for each genotype. The fold changes in expression of both *Prg*4^−/−^ and *Prg*4^+/−^ relative to *Prg*4^+/+^ were calculated separately by dividing ∆*C*_T_(*Prg*4^−/−^)/∆*C*_T_(*Prg*4^+/+^) and ∆*C*_T_(*Prg*4^+/−^)/∆*C*_T_(*Prg*4^+/+^). The fold change in gene expression of interest as it is affected by either the null or single allele expression of *Prg*4 was determined by calculating the reciprocal of the above quotients.

### 4.6. Measurement of ROS and Peroxynitrite

Reactive oxygen species (ROS), specifically peroxynitrite (ONOO^−^) and hydroxyl radical (^−^OH) production by the resected femoral head from *Prg*4 mutant mice (*n* = 5 for each genotype) were quantified using 2-[6-(4′-hydroxy) phenoxy-3H-xanthen-3-on-9-yl]-benzoic acid (HPF, Daiichi Pure Chemicals, Tokyo, Japan). Tissues were cultured in 96-well cell-culture plates (8 × 10^4^ per well) with DMEM control. The cells were incubated for 20 min at 37 °C with HPF (10 µmol/L). The fluorescent HPF activated by ROS was quantified in a fluorometer with an excitation wavelength of 490 nm and an emission wavelength of 515 nm.

### 4.7. Measurement of MitoSOX

Resected femoral heads from each genotype (*n* = 5 mice for each) were incubated in a working solution of MitoSOX Red mitochondrial superoxide indicator (M36008 Molecular Probes, Inc., Eugene, OR, USA), in DMEM. The tissues were incubated at 37 °C for 6 h, protected from light. After incubation, fluorescence was measured in a Spectra Max M2 fluorescence reader (Molecular Devices, Sunnyvale, CA, USA) with an excitation wavelength 510 nm and an emission wavelength 580 nm. ROS and MitoSOX data were normalized by cartilage weight and reported as count/mg cartilage against baseline DMEM fluorescence levels.

### 4.8. Imaging of Synoviocytes Stained with MitoSOX

Isolated *Prg*4^−/−^ and *Prg*4^+/+^ synoviocytes (1 × 10^5^ cells/mL) were added to each chamber slide, incubated at 37 °C in 5% CO_2_ incubator for 24 h. After washing with PBS, 1.0 mL of 5 µM MitoSOX™ reagent was added in Hank’s balanced salt solution (Thermo Fisher Scientific, Waltham, MA, USA) to cover the synoviocytes. Incubation occurred for 10 min at 37 °C protected from light. Cells were gently washed five times with PBS buffer at room temperature. Slides were mounted with medium compatible with fluorescence by DAPI obtained from Vector Laboratories (Burlingame, CA, USA). Imaging slides by confocal microscopy was conducted at the excitation and emission wavelength of 510 and 580 nm respectively.

### 4.9. Caspase-8 Activity Quantification

Cartilage and synovial tissue from the three genotypes were suspended in 1 mL of lysis buffer containing 50 mM Tris HCl (pH 7.4), 0.1 mM sodium orthovanadate, 50 mM sodium fluoride, 150 mM sucrose, 1 mM phenylmethylsulfonyl fluoride (PMSF), 5 mM ethylenediaminetetraacetic acid (EDTA), 5 mM ethylene glycol-*bis*(β-aminoethyl ether)-*N*,*N*,*N*′,*N*′-tetraacetic acid (EGTA), 2 µg/mL leupeptin, 2 µg/mL aprotinin, and 5 µg/mL pepstatin A. Mixtures were homogenized and microcentrifuged at 14,000 rpm for 15 min at 4 °C. The protein content of the supernatant was determined using a detergent compatible-protein assay (Bio-Rad, Hercules, CA, USA). A total of 100 µL of diluted (10 μg/mL) extract was mixed with 100 µL Caspase-Glo^®^ assay kit (Promega, Madison, WI, USA) in a 96-well plate. Briefly, wells were then gently mixed with a plate shaker at 300–500 rpm for 30 s [51] and was incubated at room temperature for 2 h. The luminescence of each sample was measured in a plate-reading luminometer (Thermo Labsystems, Thermo Fisher Scientific) with parameters of 1 min lag time and 0.5 s/well acquisition time. All experiments were performed in triplicate.

### 4.10. Tissue Processing and Hematoxylin and Eosin Staining

Following COF measurement or at the time of sacrifice, all knee joints from the in vivo study were fixed in 10% formalin (Fisher PROTOCOL™, Fisher Scientific, Waltham, MA, USA) and decalcified using a solution of 0.48 M EDTA, with adjusted pH of 7.1 with ammonium hydroxide at 4 °C for 48 h. Embedded sections were stained with hematoxylin and eosin (H&E).

### 4.11. Immunodetection of Caspase Proteases and Signal Transduction Pathways

Following SDS-PAGE electrophoresis of cartilage and synovial tissue protein extracts, and transfer to nitrocellulose, the membranes were blocked in PBS (25 mM K_2_HPO_4_ and 150 mM NaCl, PH 7.4) containing 5% *w*/*v* non-fat dried milk for 1 h at room temperature. Following three washes in PBST (PBS with 0.05% *w*/*v* Tween 20), a monoclonal antibody (1:1000 dilution with 5% bovine serum albumin (BSA) in PBST) or a polyclonal antibody (1:1000 dilution in with 5% BSA in PBST) was incubated with the membrane for 20 h at 4 °C. The monoclonal antibodies individually used were anti-caspase 6 EPR18043 (#185645, Abcam, Cambridge, MA, USA), anti-caspase 8 EPR17366 (#181580 Abcam), anti-*S*-nitrosocysteine HY8E12 (#94930 Abcam), anti-caspase 9 EPR18107 (#202068 Abcam), and anti-PARP E51 (#32064 Abcam). The polyclonal anti-bodies individually used were caspase 12 (#62484, Abcam), anti-ERK1,2 phospho (#47339 Abcam), and anti-caspase-3 (#9661, Cell Signaling Technology, Danvers, MA, USA). Following three washes in PBST, the membranes were incubated with IRDye goat anti-mouse or goat anti-rabbit IgG at 1:10,000 dilution for 1 h at room temperature with shaking, and protected from light. After incubation, the membranes were washed three times in PBST and recorded by an Odyssey Image Studio v5.2 (LI-COR Biotechnology, Lincoln, NE, USA).

### 4.12. Blot Imaging and Densitometric Analysis

The blots were imaged with LI-COR Odyssey imaging system with 800 or 700 CW according to manufacturer’s instruction. NIH image J (https://imagej.nih.gov/ij/) was used for chemiluminescent band analysis (NIH, Bethesda, MD, USA). Lanes were compared to a β-actin standard and densitometrically normalized [52] to an anti-β actin monoclonal antibody (#926-42210 LI-COR Biosciences, Lincoln, NE, USA).

### 4.13. Immunohistochemistry for Activated Caspase-3 and Nitrosylated Cys163 Caspase-3

Thin coronal sections (6 µm) were taken for histological analysis of caspase-3 activation of chondrocytes in the femoral condyles and tibial plateaus. Sections were heated to 60 °C for 30 min, deparaffinized and hydrated in three changes of xylene and serial alcohol. Antigen retrieval was performed using a pepsin solution (Thermo Fisher Scientific). A rabbit polyclonal antibody against active caspase-3 antibody 9661 (Cell Signaling Technology) or anti-*S*-nitrosocysteine HY8W12 (Abcam) at 1:000 dilution were added to separate sections and incubated at 4 °C overnight. After three washes with PBS, the sections were incubated with Cy3 goat anti-rabbit IgG (Life Technologies, Molecular Probes^®^, (Thermo Fisher Scientific) at 1:000 dilution for 1 h at room temperature, protected from light. The sections were washed five times using PBS, counterstained using Vectashield mounting media with DAPI (Vector Laboratories Inc, Burlingame, CA, USA).

### 4.14. Quantification of Active Caspase-3 and Nitrosylated Cys163 Caspase-3

Images were captured with a 20× objective, using a Roper Scientific Photometrics CoolSNAP HQ2 monochrome camera (Photometrics, Tucson, AZ, USA) and an Eclipse 90i microscope (Nikon Instruments, Avon, MA). Fluorescent images were thresholded uniformly to reduce background auto-fluorescence and to adjust DAPI signal using Adobe Photoshop CS5 software (Adobe Systems Inc., Waltham, MA, USA). Cells that probed positive for active caspase-3, and the total number of cells in femoral condyles and tibial plateau above the tide mark were counted manually using Image-Pro Premier software v9.1.4 (Media Cyberkinetics, Bethesda, MD, USA). In separate sections the same procedure was repeated for cells that probed positive for nitrosylated Cys163 caspase-3. The number of immune positive chondrocytes was counted from 200 DAPI stained chondrocytes in five tibial and femoral locations in adjacent sections. These ratios were averaged from five *Prg*4^+/+^ and five *Prg*4^−/−^ knee joints and presented as a percentage for each joint.

### 4.15. Statistical Analyses

Comparisons across experimental conditions on COF was performed using mixed model analysis of variance (SAS, PROC MIXED). The model consisted of one across-subject factor, condition (HSL, sham and control groups) and one within-subject factor, knee joints (injected vs. contralateral). For the control group in which neither knee joint was injected, the within-subject factor represented right vs. left knee joint. A similar mixed model was used to compare experimental conditions on the percentage of caspase-3 activated chondrocytes. This analysis was limited to the HSL and sham conditions, as Caspase-3 was measured in only one knee joint in control animals. For both outcomes measures, partial F-tests were used to evaluate differences across knee joints (i.e., injected vs. non-injected) within each experimental condition and linear contrasts were used to perform pairwise comparisons among conditions on differences between injected and non-injected knee joints. A two-sample t-test was used to compare the percentage of activated caspase-3 chondrocytes in the injected knee joint of the sham group to the unilateral knee joints measured in the control group. In addition, Wilcoxon signed rank test was used to compare the percentage of immunopositive chondrocytes displaying activated caspase-3 and nitrosylated Cys163 within the same joints of *Prg*4^+/+^ and *Prg*4^−/−^ mice. The ROS and MitoSOX counts/mg cartilage, relative PN production, caspase-8 Glo activity and Western blot densitometry across *Prg*4 genotypes were compared using a one-way ANOVA with pairwise comparisons performed using Tukey’s HSD procedure. All data were reported as mean ± standard deviation (SD). Statistical analyses were performed using Prism 6 software (GraphPad, La Jolla, CA, USA) and SAS statistical software Version 9.4 (SAS Institute, Cary, NC, USA) with statistical significance determined based on α = 0.05.

## 5. Conclusions

Deficiency of PRG4 at the articular surface results in dyscrasia of mitochondria within chondrocytes characterized by activation of intrinsic apoptosis pathways resulting in caspase-3 activation. This catabolic state is partly reversed by the intra-articular administration of PRG4 which lessens the exaggerated mechanical shear induced by cartilage surface friction in the absence of PRG4. Activity of caspase-3 is likely counterbalanced by the nitrosylation of caspase-3 vis-à-vis by peroxynitrite, which may arrest apoptosis and produce a stressed chondrocyte phenotype. This may explain why cartilage from patients with CACP, and from *Prg*4^−/−^ mice, appears abnormal but has not resulted in complete cellular and tissue loss. The phenomenon of friction-induced chondrocyte stress may also play out in the transient loss of PRG4 in traumatic joint injuries and thus play a role in the inciting arthrosis responsible for OA years later.

## Abbreviations

CACP	Camptodactyl-arthropathy-coxa vara-pericarditis
ROS	Reactive oxygen species
COF	Coefficient of friction
OA	Osteoarthritis
IA	Intra-articular
HSL	Human synoviocyte lubricin (PRG4)
PN	Peroxynitrite
O^−^_2_	Superoxide
NO	Nitric oxide
mRNA	Messenger RNA
ONOO^−^	Peroxynitrite
^−^OH	Hydroxyl radical
DAPI	4,6-Diamidino-2-phenylindole, dihydrochloride
EDTA	Ethylenediaminetetraacetic acid
EGTA	Ethylene glycol-*bis*(β-aminoethyl ether)-*N*,*N*,*N*′,*N*′-tetraacetic acid
PMSF	Phenylmethylsulfonyl fluoride
H&E	Hematoxylin and eosin
*Prg4*	Murine lubricin gene
PRG4	Purified lubricin protein (Proteoglycan 4)
